# Oral administration of alanyl-glutamine and glutamine improve random pattern dorsal skin flap survival in rats 

**DOI:** 10.22038/IJBMS.2018.29629.7153

**Published:** 2018-08

**Authors:** Mojtaba Karimipour, Morteza Hassanzadeh, Masoumeh Zirak Javanmard, Gholamhossein Farjah

**Affiliations:** 1 Department of Anatomy, Faculty of Medicine, Urmia University of Medical Sciences, Urmia, Iran; 2 Neurophysiology Research Center, Department of Anatomy, Faculty of Medicine, Urmia University of Medical Sciences, Urmia, Iran

**Keywords:** Alanyl-glutamine, Flap, Glutamine, Rats, Survival, VEGF

## Abstract

**Objective(s):**

Skin flap necrosis is the most common postoperative side effect in reconstructive surgeries. Glutamine (GLN) has been shown to accelerate wound healing process. The purpose of this study was to evaluate the effects of GLN either in free form or in the dipeptide form along with L- alanyl (Ala-GLN) on random skin flaps survival in rats.

**Materials and Methods:**

Dorsal skin flaps with caudal bases (8 ×2 cm) were established in 24 adult male Wistar rats. Then, the animals were randomly assigned into 3 groups (n=8). Control, GLN (0.75 g/kg) and Ala-GLN (0.75 g/kg). All groups administrated orally 24 and 6 hr before flap elevation and continued repeatedly daily until 7 days postoperation. The flap survival rate and vascular density using histological analysis were evaluated. Vascular endothelial growth factor (VEGF) by immunohistochemical method was determined.

**Results:**

Seven days after surgery, the mean surviving area in the GLN and Ala-GLN groups were significantly greater than in the untreated control group (*P*<0.001). Furthermore, in comparison with the control group, the number of blood vessels and VEGF-positive cells in treated groups with GLN and Ala-GLN were significantly higher. However, no significant differences were observed between treated groups with GLN and Ala-GLN.

**Conclusion:**

The findings from this study indicate that oral administration of GLN in free form or in the dipeptide (Ala-GLN) could promote neovascularization and improve skin flap survival in rats.

## Introduction

Random skin flaps are widely used in plastic and reconstructive surgery to repair large acquired or congenital defects. It is a surgical method for skin damages, especially when there is a need for massive reconstruction of complex anatomic structures, such as reconstruction of breast and covering on amputation stump (1). Unfortunately, necrosis as an unwanted complication is frequently observed in distal area of flap tissues after surgery. The main causes of necrosis are ischemia, inadequate blood flow and impaired venous drainage (2). This major complication can elongate hospital stay and increase the cost of treatment and it may even lead to a loss of patient confidence in medical doctor (3). To address this issue, various therapeutic approaches have been applied to reduce ischemia reperfusion (IR) induced tissue damage by using pharmacological agents (4), bone marrow mesenchymal-derived stem cells (5), and devices such as laser (6) and low dose radiation (7). Nevertheless, neovascularization, new blood vessel formation, in flap tissues has been remained as a serious unsolved complication.

IR damage is one of important cause of morbidity after trauma. The involved mechanisms in IR injury are multifactorial and one solution way is supplementation of drug or agents to decrease components of IR damage, such as complement activation, neutrophil infiltration, reactive oxygen species (ROS) and inflammation (8).

Glutamine (GLN) is the most abundant free amino acid in plasma and stored in several tissues including lung and skeletal muscle. GLN is a nonessential amino acid, but becomes conditionally essential in conditions of severe stress in which intracellular GLN levels decrease more than 50% and plasma concentration by 30% (9). GLN synthesized in the intestine by protein digestion, but studies have shown that only small amounts of the GLN enters bloodstream (10). It has been proven that GLN levels in plasma and skeletal muscle decreases during sepsis, surgery, injury and burns (11). In stressful status, the production of GLN impairs by body. Chamney *et al*. have demonstrated that in spinal cord injury cases in comparison with non-injured subjects, plasma glutamine concentration was reduced by 54% (12). Previous study has shown that GLN has positive effects against IR injury (8). Other studies also have demonstrated that pre-treatment with GLN decreased oxidative stress and improved cell survival and had a protective effect against damage to organs after IR (13, 14). Su Kim *et al* reported that GLN alleviated cerebral ischemic injury in cardiac arrest model of rats (15). GLN alleviates inflammation. In a recent study it has been shown that GLN reduce levels of IL6 and TNFα in muscle of mice model of spinal cord injury (12). 

It is known that GLN has a critical role in wound healing. A recent study showed that oral administration of GLN enhances wound healing process by acting on various stages of wound healing, including collagen synthesis, wound contracture, and epithelialization (16). Moreover, GLN significantly decreases cisplatin-induced genotoxicity in bone marrow cells in rats (17). Other animal studies have reported the positive effects of GLN on healing of burn wounds (18).

The results from studies that used GLN in its free form are controversial and oral administration with GLN in the dipeptide form, such L- alanyl -L-glutamine (Ala-GLN) seems to be more effective and provides an alternative way to elevate the level of GLN in the body in stressful situations (19, 20). It has been reported that oral administration of GLN in form of Ala-GLN is effective in alleviating oxidative stress and proinflammatory responses induced by endotoxemia in mice (21).

There is no report about the effect of GLN on improvement of survival rate in random skin flap. Hence, in this work, we tested the hypothesis that oral administration with GLN in forms of free or Ala-GLN could prevent necrosis and improve recovery in rats subjected to random skin flap surgery.

## Materials and Methods

***Animal and groups***

Twenty-four male Wistar rats, 10-12 weeks old and weighing 230-250 g were used in this study. After the adaptation period, they have been assigned randomly to three groups, each containing eight rats.

Control group; received 0.5 ml/day water by gastric gavage.

Glutamine group (GLN); received 0.75 g/kg/day GLN (Sigma Chemical Co) 24 and 6 hr before flap creation until 7 days after surgery by gastric gavage.

Alanyl –glutamine group (Ala-GLN); received 0.75g/kg/day alanyl -GLN (Sigma Chemical Co) 24 and 6 hr before flap creation until 7 days after surgery daily by gastric gavage.

The dose of GLN administration was, according to previous study (22). After flap surgery, the animals were housed in individual cages and they were accessed to water and food *ad libitum* throughout the experiment. The study was performed in accordance with Guide for the Care and Use of Laboratory Animals and was approved by Urmia University of Medical Sciences Ethics Committee.

***Skin flap surgery***

The animals were anesthetized with an intraperitoneal injection of a solution of ketamine (60 mg/kg, Alfasan, The Netherlands) and xylazine (10 mg/kg, Alfasan, The Netherlands). After inducing deep anesthesia, the rats were kept in prone position and then their backs were shaved with electric clippers. After skin disinfecting with a povidone iodine solution, a caudally-based 8 × 2 cm random pattern skin flap were created in the dorsum of each rat according to the procedure described by McFarlane. After elevation of the flaps, they were replaced to original position and immediately sutured back using 4-0 silk (23).

***Flap survival assessment***

On the seventh day after flap surgery, the rats were anesthetized and the survival and necrosis areas were demarcated on a transparent paper and then cut the paper and weighed using a precision scale. By using the below formula, the percentage of survival rate of each flap was determined (24). 

Percentage of skin flap survival=Weight of survival area Total weight of paper×100

The living flap tissues were identified by gross observation and to the touch were warm and soft but, the necrotic areas were dark in color, stiffness, and hairless.

***Histological evaluations***

*Blood vessels density*

Seven days after flap creation, the rats were euthanized with high doses of anesthetic substance (ketamine and xylazin) and then, the skin tissue samples were collected from the same place of the surviving part of the flap. The skin samples were fixed at 10% buffered formalin for 24 hr, embedded in paraffin wax and then, the longitudinal sections with a thickness of 6 µm were prepared and stained with hematoxylin and eosin (H&E). For assessment of blood vessels density, the vessels were counted in five randomly selected fields using a light microscope at 100× magnification (24).

*VEGF immunostaining*

To evaluate the VEGF expression levels in flap tissues using immunohistochemically method, the tissue sections from paraffin-embedded blocks were dewaxed and rehydrated using xylene and ethanol and then, washed with phosphate buffered saline (PBS) solution. The samples were incubated in 10 mM sodium citrate at 36 for 30 min in order to perform antigen retrieval. Then, the sections were treated with H_2_O_2_ (3%) to block endogenous peroxidase and then washed with PBS. Next, the slides were incubated with primary anti-VEGF antibody solution (Abcam, 1:200 dilution overnight at 

4 °C). 4. After washing with PBS, secondary antibody was added to slides for 45 min at 37 , and washed. Then, the slides were treated with diaminobenzidine solution for 10 min at room temperature for color development. Next, the slides were counterstained with hematoxylin and were observed by light microscope. The VEGF-positive brown color cells were counted in two fields at 400× magnification by at least two persons blinded to treatment (25). 

***Statistical analysis***

The data were presented as mean ± standard deviation (SD) and all statistical analysis was performed by SPSS version 16 for Windows (SPSS, Chicago, USA) using analysis of variance (One Way ANOVA), with Tukey`s *post hoc* test to compare the means. *P*-value of 0.05 was considered as significant. 

## Results

***Flap survival***

**Figure 1:**
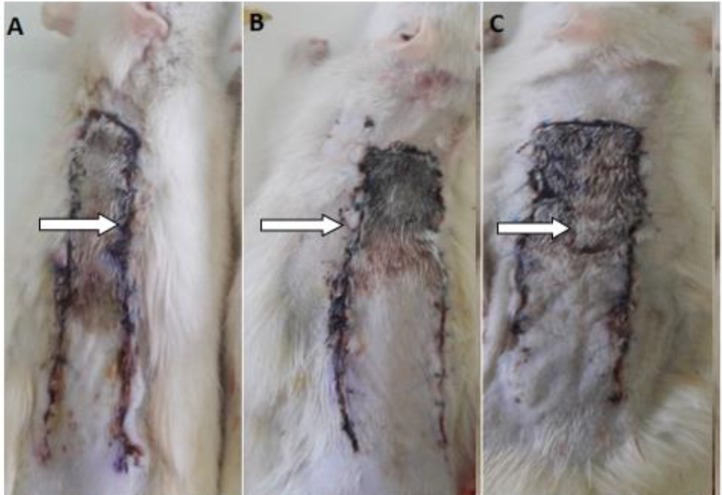
Digital photographs of random –pattern dorsal skin flaps 7 days after surgery.(A) control group; (B) glutamine group; and (C) alanyl-glutamine group.The arrows showing the necrotic areas

**Figure 2:**
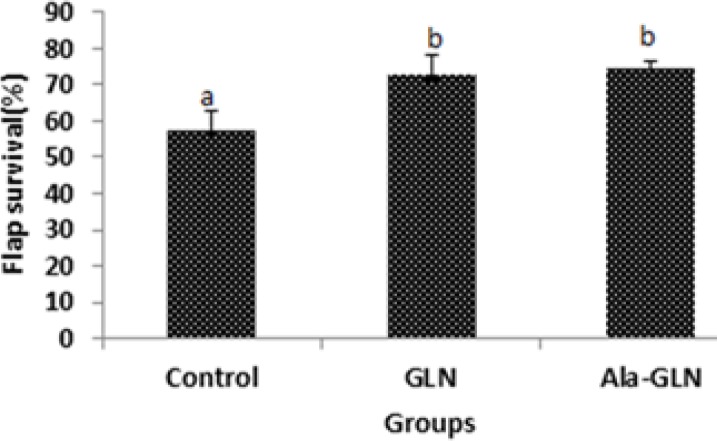
The mean ± SD of the percentage of flap survival in different groups

Figure 1 shows the areas of survival and necrosis of flaps from the different groups on seventh day after flap surgery. The mean ± SD of flap survival percentage were 72.61±5.79 in the GLN group and 74.32±2.45 in the Ala-GLN group, which were significantly higher than the control group (57.7±5.29) (*P*<0.001). However, there was no statistical significant difference between the GLN and Ala-GLN groups (Figures 1, 2). 

b Significantly different from control group (*P*<0.01)

**Figure 3:**
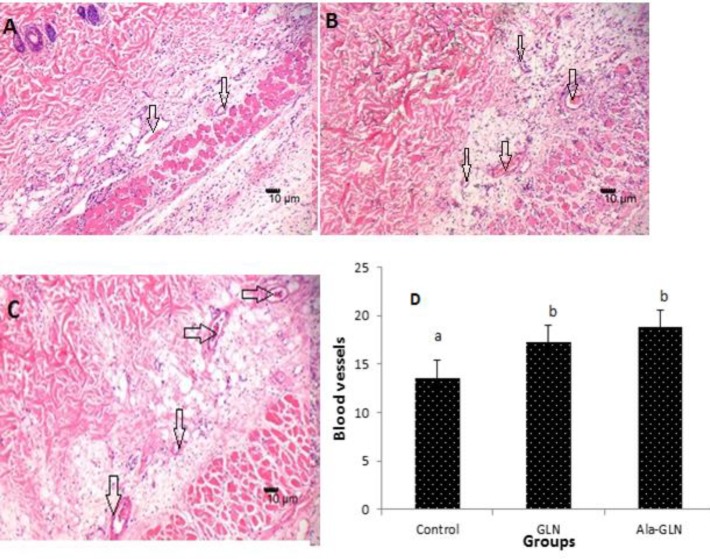
Blood vessels in the flap tissue of different groups on seventh postoperative day in the slides stained with H&E. (A) Control group; (B) Glutamine (GLN) group; and (C) Alanyl-glutamine (Ala-GLN ) group. The arrows showing vessels. Original magnification 100Χ; (D) The mean ± SD of blood vessel number in groups

b Significantly different from control group (*P*<0.05)

**Figure 4:**
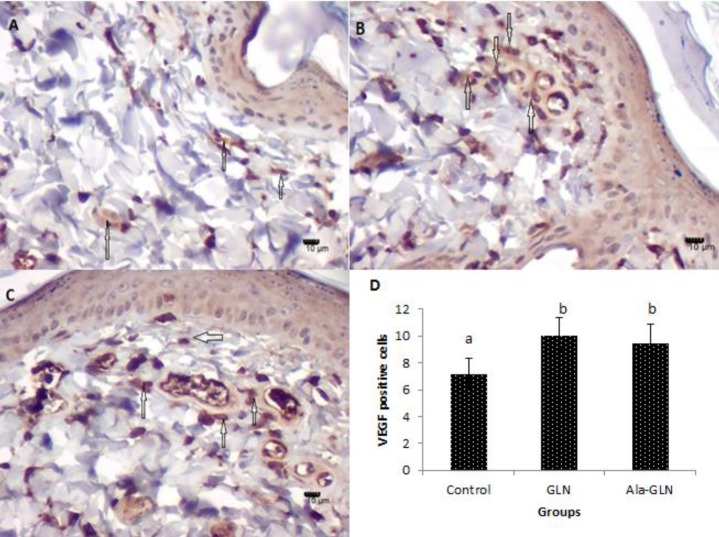
Photographs of Immunohistochemical staining for VEGF in flap tissue in different groups. (A) Control group; (B) Glutamine group; and (C) Alanyl-glutamine group. The arrows showing VEGF positive cells. Original magnification 400Χ. (D) The mean ± SD of VEGF positive cells at seven days after flap creation

b Significantly different from control group (*P*<0.01)

***Histological findings***

*Vascular density in flaps*

New blood vessels formation in survival area of the skin flaps in different groups are shown in Figure 3. Seven days after flap creation, the mean ± SD of vascular density were 13.57 ± 2.87, 17.28 ± 2.69, and 18.85 ± 1.67 in the control, GLN, and Ala-GLN groups respectively. There was a significant increase in the treated groups with GLN and Ala-GLN groups (*P*<0.05) when compared to the control group. The mean number of blood vessel formation in the Ala-GLN group was higher than GLN group, but it was not significant.

*VEGF levels in flaps*

The mean±SD numbers of the VEGF-positive-cells in flap tissues on seventh day postoperation were 10 ± 1.41 in the GLN group and 9.5 ± 1.37 in the Ala-GLN group, which were significantly (*P*<0.01) higher than the control group (7.16 ± 1.16). There was no statistical significant difference between GLN and Ala-GLN groups (Figure 4).

## Discussion

In the current study, we set out to evaluate whether oral administration of GLN in free form and in a form of Ala-GLN would improve survival rates in random pattern skin flaps. Our findings indicated that decreased necrosis of skin flaps in rats could be achieved by GLN supplementation.

Random skin flaps are commonly used to repair massive soft tissue injuries. But, postoperative ischemia and necrosis are common side effects observed between 25 to 50% of the total area of the flap particularly at its distal part due to low blood flow (2, 26, 27). It has been documented that IR injury is the major cause of skin flap necrosis, which is related to several pathological processes such as, ischemia, free radical damage, leukocyte adhesion, and inflammatory mediators production following tissue and blood vessels damage (28). Thus, improving the survival of skin flaps mainly related to neovascularization acceleration (29). Our results demonstrated that vessel density in animals supplemented either with GLN in free form or with Ala-GLN form was increased. This finding was proven by the histological evaluation and was also apparent to the naked eye. The increase in vessel density is the major cause of increased flap survival in the groups supplemented with GLN. Findings obtained from the present study suggest that oral administration of GLN may accelerate neovascularization and promote microcirculation in ischemic flaps by increasing VEGF expression and ultimately improving flap survival. The details of the involved mechanisms need to be further studied.

In the present study, we have shown that 24 and 6 hr GLN pretreatment until 7 days postoperation can diminish IR associated with skin flap necrosis in rats. To our knowledge, this is the first study dealing with beneficial effects of GLN supplementation in viability of skin flaps. We have also shown that GLN in both free and dipeptide (Ala-GLN) forms has capability to improve flap viability in comparison with control group, but there was no significant difference between groups received GLN in free form and Ala-GLN form.

During stressful conditions, GLN accessibility to the body decrease. This reduction in GLN is along with reducing of glutathione (GSH) synthesis, because GLN is the precursor of GSH. And it has been shown that plasma GLN concentration is the major resource of GLN for GSH producing by erythrocytes. GSH is a potent antioxidant and has the potential to neutralize ROS by reducing lipid peroxidation (30-32). Furthermore, the antioxidant properties of GLN via GLN-GSH axis is able to reduce inflammatory response mediated by TNFα and IL1β (33). 

In this study, we compared beneficial effects of GLN in free form with Ala-GLN on skin flap viability in rats. According to the previous reports, alanyl in the Ala-GLN can spare GLN metabolism and facilitate GLN availability to damaged tissue (21, 34). It is believed that GLN is more effective and stable in the form of dipeptides and is a suitable way to compensate for the concentration of GLN in the body (19, 21) but, our findings did not confirm it, as the findings showed that Ala-GLN in comparison with GLN had a better effect on flap viability without significant difference. Thus, the data of the current study proposed that GLN has the same effects on flap survival when compared with Ala-GLN. Previous reports have shown that Ala-GLN are usually preferred, because GLN in the Ala-GLN is metabolized more slowly by enterocytes and this makes it possible that GLN concentration in the plasma is rapidly increasing. This effect has been associated with Pept-1, which is located in the intestinal membrane and has the ability to transport Ala-GLN in the gut of humans and animal (35). In the current study, we did not investigate the effects of L-alanyl alone on flap viability. Thus, from this study, we cannot explain whether this effect is due to GLN or alanyl. However, based on the our findings, we suggest the presence of Ala-GLN cannot improve the survival of the flap significantly more than when compared with GLN alone.

 Prior studies documented that citrulline and arginine are two end products of GLN metabolism in the intestine (36, 37). In a recent report it was revealed that administration of L-arginine (precursor of nitric oxide) to rats improved survival rate of skin flap (38). 

The results of the present study provide the first evidence that daily GLN supplemental in form of GLN or Ala-GLN form both can significantly improve flap survival rate in comparison with control in rats.

As we showed, flap necrosis in the rats supplemented with GLN significantly reduced. This beneficial effect of GLN administration has also been reported in the rodent model of wound healing. Furthermore, there is no evidence to support the major adverse side effects of GLN supplementation in human healthy (39).

Previous studies have shown that GLN supplementation improves body recovery from various stressful condi-tions such as, endotoxaemia, IR injury and spinal cord injury. These improved recoveries have been reported to be related to increase in levels of heat shock proteins (HSP) in the tissue (12). HSP or stress proteins, are proteins which produced by cells in response to exposure to stressful conditions. Previous experimental studies have revealed that supplementation of GLN attenuates cerebral ischemia injury through increasing HSP-72 and HSP-25 (15). Altogether, many previous studies suggest that the mechanism of GLN in reducing organs IR damage is partly due to HSP expression, which in turn has anti-inflammatory and anti-apoptotic effects (40). In the present study, we can assume that the GLN administration, possibly has induced HSP expression in the location of skin flaps, but still to be determined.

Our study is not without limitation. We did not measure levels of HSP, inflammatory mediators and oxidative stress. One suggestion for a future study is to determine the relationship between flap survival rate and HSP expression following GLN supplementation.

As mentioned earlier in literature, GLN has other many pharmacological effects, including anticancer, anti-bacterial, antiulcer, anti-apoptotic, hepatoprotective and wound healing. In a prior study it was shown that oral administration of GLN increased hydroxyproline content in wound, which reflects increased collagen synthesis, and is an important marker in the process of wound healing (16). Furthermore, administration of exogenous GLN accelerated epithelialization and significantly declines the period of wound contraction (16). Increased new blood vessel formation in the present study is in accordance with above mentioned study. The mechanisms by which GLN promote neovascularization is not fully elucidated. In the present study, we showed that oral administration of GLN increased VEGF expression in a rat skin flap model. VEGF as an important proangiogenic molecule in the skin is produced by keratinocytes, mast cells, and macrophages in damaged skin (41, 42). It has been reported that VEGF accelerates the neovascularization process and increases the number of blood vessels in the ischemic region (43, 44). As it was stated above, oral administration of GLN increased the VEGF expression in skin flaps. Thus, it is possible that GLN through this mechanism increases the new blood formation and ultimately improves the survival rate of the flap. 

## Conclusion

The findings from this study indicate that oral administration of GLN, either in the free form or in the form of dipeptide along with L-alanyl (Ala-GLN) through promoting neovascularization, effectively improves the survival rate of random skin flaps in rats. However, further studies are needed to determine the mechanisms of GLN for alleviating the necrosis rate of dorsal random skin flaps.
